# Different Toxicity Mechanisms for Citrinin and Ochratoxin A Revealed by Transcriptomic Analysis in Yeast

**DOI:** 10.3390/toxins8100273

**Published:** 2016-09-22

**Authors:** Elena Vanacloig-Pedros, Markus Proft, Amparo Pascual-Ahuir

**Affiliations:** 1 Department of Biotechnology, Instituto de Biología Molecular y Celular de Plantas, Universidad Politécnica de Valencia, Ingeniero Fausto Elio s/n, 46022 Valencia, Spain; mevaped@etsia.upv.es; 2 Department of Molecular and Cellular Pathology and Therapy, Instituto de Biomedicina de Valencia IBV-CSIC, Jaime Roig 11, 46010 Valencia, Spain

**Keywords:** Ochratoxin A, Citrinin, Transcriptome, *Saccharomyces cerevisiae*, mycotoxins, oxidative stress, dose response

## Abstract

Citrinin (CIT) and ochratoxin A (OTA) are important mycotoxins, which frequently co-contaminate foodstuff. In order to assess the toxicologic threat posed by the two mycotoxins separately or in combination, their biological effects were studied here using genomic transcription profiling and specific live cell gene expression reporters in yeast cells. Both CIT and OTA cause highly transient transcriptional activation of different stress genes, which is greatly enhanced by the disruption of the multidrug exporter Pdr5. Therefore, we performed genome-wide transcription profiling experiments with the *pdr5* mutant in response to acute CIT, OTA, or combined CIT/OTA exposure. We found that CIT and OTA activate divergent and largely nonoverlapping gene sets in yeast. CIT mainly caused the rapid induction of antioxidant and drug extrusion-related gene functions, while OTA mainly deregulated developmental genes related with yeast sporulation and sexual reproduction, having only a minor effect on the antioxidant response. The simultaneous exposure to CIT and OTA gave rise to a genomic response, which combined the specific features of the separated mycotoxin treatments. The application of stress-specific mutants and reporter gene fusions further confirmed that both mycotoxins have divergent biological effects in cells. Our results indicate that CIT exposure causes a strong oxidative stress, which triggers a massive transcriptional antioxidant and drug extrusion response, while OTA mainly deregulates developmental genes and only marginally induces the antioxidant defense.

## 1. Introduction

Mycotoxins are small toxic molecules produced by a great variety of microorganism, which encompass several classes of secondary metabolites with no common chemical structure or mode of action [1]. These harmful natural products of molds contaminate food and feed worldwide with appalling economic consequences, since they affect most of the staple food crops such as maize, wheat and rice [2,3]. Beyond the economic losses, mycotoxins have a severe impact on human wellbeing [4]. Their toxicological properties and possible health effects have been extensively studied and related to some diseases, although it is certainly difficult to demonstrate the link between toxin exposure and the onset of symptoms in most cases. Mycotoxins are released by some fungi in nature for unclear reasons, and although it is widely accepted that the synthesis and secretion of toxins mediate pathogen virulence of microorganisms in plants, the molecular targets and strategies to achieve it remain to be determined in the case of mycotoxins [5]. Considerable efforts have been made to comprehend the molecular mechanisms of mycotoxins to cause cell damage and toxicity [6,7,8]. Although it is desirable to understand the molecular basis of mycotoxin action in whole animals, these approaches are often difficult because the dose-effect relation depends on many different parameters [7]. As an alternative, the fundamental modes of toxicity for individual mycotoxins can be efficiently revealed in cell cultures of lower eukaryotic cells such as yeast.

Ochratoxins are a small group of mycotoxins produced by *Aspergillus* and *Penicillium* species, with ochratoxin A (OTA) as the principal compound, found in a very wide range of raw and processed food [9]. OTA is nephrotoxic, carcinogenic, and a potent teratogen when tested in different mammalian models, and thereby is a potential risk to human health [10]. Several authors support that the mode of action of OTA implies the formation of covalent DNA adducts [11,12,13] and the increase of reactive oxygen species [14,15], hence these activities could explain the genotoxic and mutagenic activity of OTA. The co-occurrence of OTA with citrinin (CIT), another mycotoxin, has been often reported [16,17]. CIT is produced by filamentous fungi of the genera *Penicillium*, *Aspergillus* and *Monascus*, and contaminates the same staple foodstuffs as OTA [18]. Fungi such as *Penicillium verrucosum* are able to produce both OTA and CIT, however, different environmental conditions might favor the production of one mycotoxin over the other [19,20,21]. Much less is known about the toxicity mechanisms of CIT, however, it has been shown to be an efficient nephrotoxin as well [22]. Several groups have contributed to the identification of possible molecular mechanisms of CIT toxicity, finding, among other consequences, the increase of oxidative stress in connection with alterations of mitochondrial function, and induction of apoptosis [23,24,25,26,27,28,29,30,31]. It has been proposed that the co-occurrence of both toxins results in synergetic effects, however no clear conclusions have been reached [32,33].

Gene expression analysis has become a valuable tool to decipher molecular mechanisms in response to toxic agents, including mycotoxins [34], and the yeast model is particularly important in toxicogenomic studies [35]. Recent transcriptomic approaches with OTA have been performed using different cell lines and mammalian model systems [36,37,38,39]. A comparison of the genomic data does not yield a uniform pattern of deregulated genes, and it is striking that DNA damage response genes are not generally highlighted by these omics approaches [40]. It seems that the variability of the OTA-induced transcriptomic response might be a consequence of the range of experimental conditions as well as the cellular context [40]. In contrast to OTA, genomic profiling data for CIT treatment are scarce, however, the application of yeast microarray approaches has identified the antioxidant defense as one of the primordial manners of detoxification upon CIT exposure [41]. The transcriptional response to mycotoxins is likely to be transient and dose dependent, therefore any transcriptomic assay is further complicated by the selection of the optimal induction conditions. Actually, in vivo recording of transcriptional activity in *Saccharomyces cerevisiae* shows a transient dose–time dependent response to CIT treatment [28].

Given that OTA and CIT are co-occurring toxicological threats in the food chain and that both overlapping and divergent mechanisms of toxicity have been proposed for both mycotoxins, we aim here to compare the immediate transcriptomic response to OTA and CIT, applied either separately or simultaneously. We use an optimized yeast system, where the optimal time point and dose for each mycotoxin has been adjusted according to live cell gene expression reporters and where the signal intensity has been largely increased due to the deletion of the principal toxin exporter Pdr5. We identify largely exclusive patterns of gene deregulation for CIT and OTA, with oxidative stress defense genes specifically activated by CIT and cell differentiation and developmental genes specifically activated by OTA.

## 2. Results

### 2.1. Gene Expression Profiles of Stress Response Genes upon CIT and OTA Exposure

**Figure 1:**
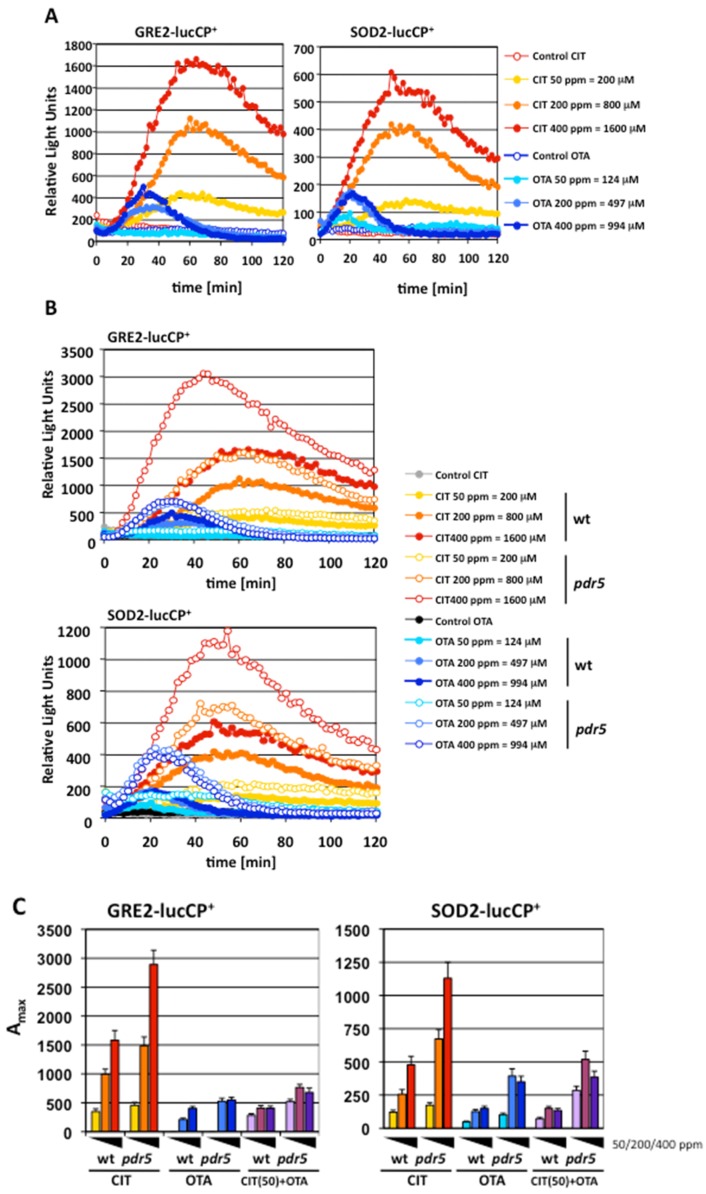
Ochratoxin A (OTA) and citrinin (CIT) activate stress gene expression independently and with different dose response profiles. (**A**) OTA and CIT induction of the stress-activated genes *GRE2* (methylglyoxal reductase) and *SOD2* (superoxide dismutase). Live cell reporter fusions with destabilized luciferase were used in yeast wild type cells and the induction of both genes was measured in real time upon the indicated mycotoxin doses. (**B**) The deletion of the Pdr5 multidrug exporter increases the transcriptional response to both OTA and CIT. The expression profiles for the *GRE2* and *SOD2* genes are compared for wild type and the *pdr5* deletion mutant upon the indicated mycotoxin doses. (**C**) OTA and CIT do not activate stress gene expression in a synergistic manner. The dose response profiles of (**A**) and (**B**) are represented here as the maximal activity (A_max_) for each mycotoxin dose. Additionally (purple columns at the right of each plot), a constant concentration of CIT (50 ppm = 200 μM) was combined with growing concentrations of OTA (50 ppm = 124 μM; 200 ppm = 497 μM; 400 ppm = 994 μM) as indicated. All gene expression experiments were performed on three independent culture aliquots; the Standard Deviation was <15%; error bars are not included in the graphs in order to make the figure clearly visible.

We have previously shown that live cell reporter fusions in yeast are valuable and quantitative tools to characterize the acute transcriptional adaptation to CIT [28]. Here, we extend these studies to compare the impact of CIT and OTA on the induction of different stress-inducible genes. We used fusions of the oxidative stress-inducible *SOD2* (mitochondrial manganese superoxide dismutase) promoter and the general stress-inducible *GRE2* (methylglyoxal reductase) promoter with destabilized luciferase as sensitive live cell reporters. Dose-dependent analyses revealed a transient gene expression profile for both reporter genes, upon treatment with CIT and OTA (Figure 1A). Both mycotoxins induced gene expression very rapidly within minutes, indicating that CIT and OTA are readily taken up by yeast cells. However, CIT caused a much broader transcriptional induction, which continuously increased with dose even beyond 400 ppm (1600 μM). OTA, in contrast, induced the stress-responsive reporters in a much more transient manner and to much lower absolute induction levels. Moreover, OTA-induced transcription of *GRE2* or *SOD2* was already maximal at concentrations around 200 ppm (497 μM). We next tested the effect of the loss of Pdr5 function, which is a plasma membrane multidrug transporter critically involved in CIT extrusion [28]. As shown in Figure 1B, the deletion of Pdr5 provokes an enhanced transcriptional response to both CIT and OTA treatment at different doses. We next wanted to study the level of synergy involved in the response to CIT and OTA using the same live cell gene expression reporters. Surprisingly, no evident synergistic effect on gene expression was revealed when both toxins were combined together, both in the wild type or the sensitized *pdr5* mutant strain (Figure 1C). Taken together, these results indicated that CIT and OTA had differential and independent effects on the induction of stress reporters in yeast. Thus we aimed at studying the differential induction of gene expression upon CIT and OTA exposure at the genomic level.

### 2.2. Genomic Expression Profiles upon Separated and Combined Exposure to CIT and OTA

Our previous study of specific stress promoters suggested that CIT and OTA had a different impact on gene expression. Both mycotoxins, however, activate gene transcription in a very transient manner. We wanted to take advantage of genome-wide transcription analysis in yeast to gain insights into the differential induction of gene expression triggered by the two mycotoxins. The microarray experiments were performed in the sensitized *pdr5* mutant strain and at optimized toxin concentrations and exposure times as revealed by our real time surveys upon acute CIT and OTA exposure. The transcriptomic response of yeast was determined by microarray hybridization upon separated CIT and OTA exposure (200 ppm) as well as upon the combined addition of CIT/OTA (100 ppm each). As a first approach, we identified and ranked the most upregulated genes for each toxin treatment. We applied a very stringent cutoff value and considered only the genes which were expressed more than 5-fold higher in the treated cells as compared to the untreated cells. The resulting gene lists are represented in Table 1 for CIT, in Table 2 for OTA, and in Table 3 for the combined CIT/OTA treatment.

Acute CIT exposure provoked the robust upregulation of 68 yeast genes. When classified for the most statistically relevant functional groups, we identified the response to oxidative stress as the dominant group (see Table 4). These data confirmed that CIT toxicity is fundamentally based on its capacity to generate reactive oxygen species (ROS) in cells. Specifically, genes involved in the metabolism of glutathione were preferentially expressed upon CIT exposure, indicating that the antioxidant function of glutathione was necessary to palliate the toxic effect of CIT. Additionally we identified “Drug transport” as a main CIT-inducible gene group, suggesting that the activated export of the toxin might be a major determinant for the adaptation of yeast cells to CIT.

**Figure 2:**
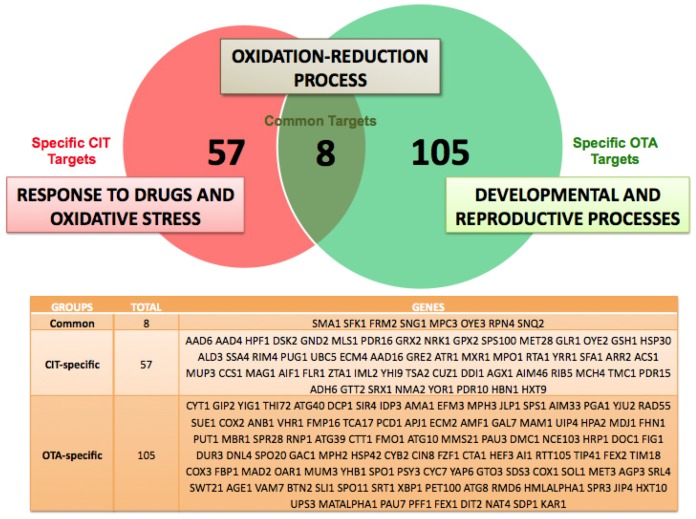
Ochratoxin A and citrinin activate largely nonoverlapping gene sets in the yeast genome. Venn diagram comparing the >5-fold induced transcripts of the yeast genome upon OTA and CIT exposure. The exclusively upregulated genes by one mycotoxin (CIT or OTA) and the commonly upregulated genes are depicted in the table. The main functional groups associated with each gene cluster are given.

For OTA exposure, we were able to identify 115 genes whose expression was at least 5-fold induced (Table 2). The analysis of the functional groups enriched in the dataset derived from OTA-treated cells revealed that the “response to oxidative stress” was retrieved with much less significance as compared to the CIT dataset. In turn, we identified yet other functional groups as most significantly upregulated by OTA, which belong to developmental processes of yeast cells and specifically to the differentiation processes of sporulation and reproduction (see Table 4). These data indicated that both mycotoxins induced different gene sets in yeast. Indeed, the comparison of the most significantly upregulated genes revealed that less than 5% (a total of only 8 genes) of the transcripts were induced commonly by either CIT or OTA as depicted in Figure 2. The subset of CIT- and OTA-responsive genes was enriched for the functional category “Oxidation–reduction process”. These results clearly showed that CIT and OTA induced largely separated gene sets in the initial adaptive phase, which suggested that both mycotoxins might have different biological effects in yeast cells. We next analyzed the transcriptomic response of yeast cells to the combined exposure of CIT and OTA. A total of 68 transcripts were significantly upregulated >5-fold under these conditions (see Table 3). The functional gene groups enriched by the combined mycotoxin treatment represented a combination of the gene functions induced in the previous experiments by the separated toxin treatment. As a result, all categories covering “oxidative stress response”, “drug transport”, “developmental processes”, and “sporulation” were significantly enriched upon the combined CIT/OTA exposure (see Table 4). Taken together, our transcriptomic survey of the response to CIT and OTA strongly supported the idea that both toxins cause distinct and separable biological responses. CIT caused a clear antioxidant response and the induction of multiple drug extrusion systems, while OTA seemed to retain a weak oxidation-related toxicity and to cause a marked deregulation of developmental genes. We wanted to further dissect these divergent toxicity effects of CIT and OTA in the yeast model.

### 2.3. Oxidative Stress is a Hallmark for CIT, but not OTA, Toxicity

**Figure 3:**
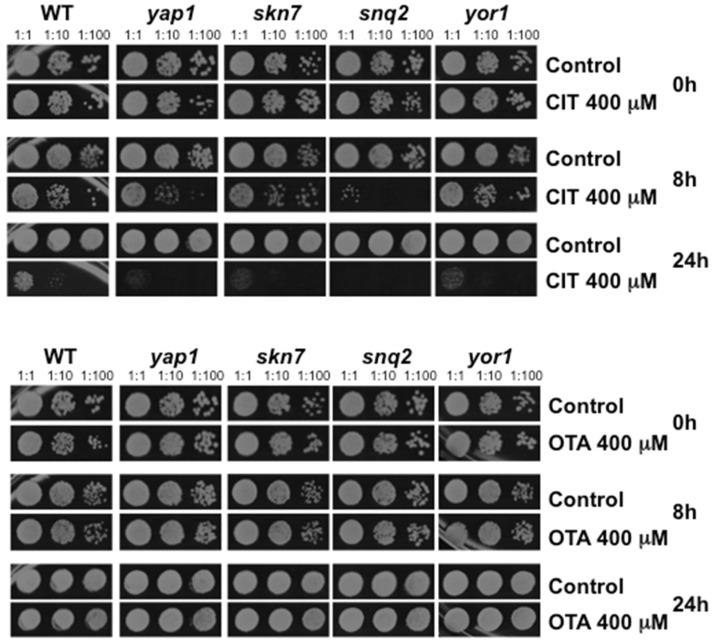
Citrinin, but not ochratoxin A, toxicity is exacerbated in mutants with a defective antioxidant response or multidrug export. The indicated yeast strains were treated or not with 400 μM CIT (upper panel) or 400 μM OTA (lower panel) for the indicated time. Serial dilutions 1:1, 1:10, and 1:100 of the yeast cultures were then assayed for survival on yeast extract peptone dextrose (YPD) agar plates without mycotoxins.

According to our genomic expression experiments, CIT caused a specific antioxidant response in yeast cells, while antioxidant genes were only weakly induced by OTA. Additionally, CIT robustly induced the expression of a total of 7 different multidrug exporters (Flr1, Atr1, Snq2, Pdr15, Pdr10, Pdr16 and Yor1), while OTA moderately activated the expression of only the Snq2 drug exporter. We therefore wanted to quantify the importance of the antioxidant response and drug transport for the resistance to CIT or OTA. We employed specific yeast mutants with a defect in the oxidative stress adaptation (*yap1*, *skn7*) or multidrug export (*snq2*, *yor1*) and tested their resistance to CIT or OTA in comparison to wild type cells. As shown in Figure 3, the lack of the principal transcriptional activator of the oxidative stress defense Yap1 or of the multidrug transporter Snq2 rendered yeast cells hypersensitive to CIT, but not OTA. This sensitivity was observed after 8 h of toxin treatment. The deletion of a second transcription factor involved in the antioxidant response, Skn7, or an alternative multidrug exporter, Yor1, resulted in a weaker sensitivity phenotype exclusively in the case of CIT, which was observed after a prolonged toxin treatment (24 h). These data indicated that the antioxidant defense and the activated toxin export are key features for CIT detoxification, which are dispensable for the cellular defense against OTA.

**Figure 4:**
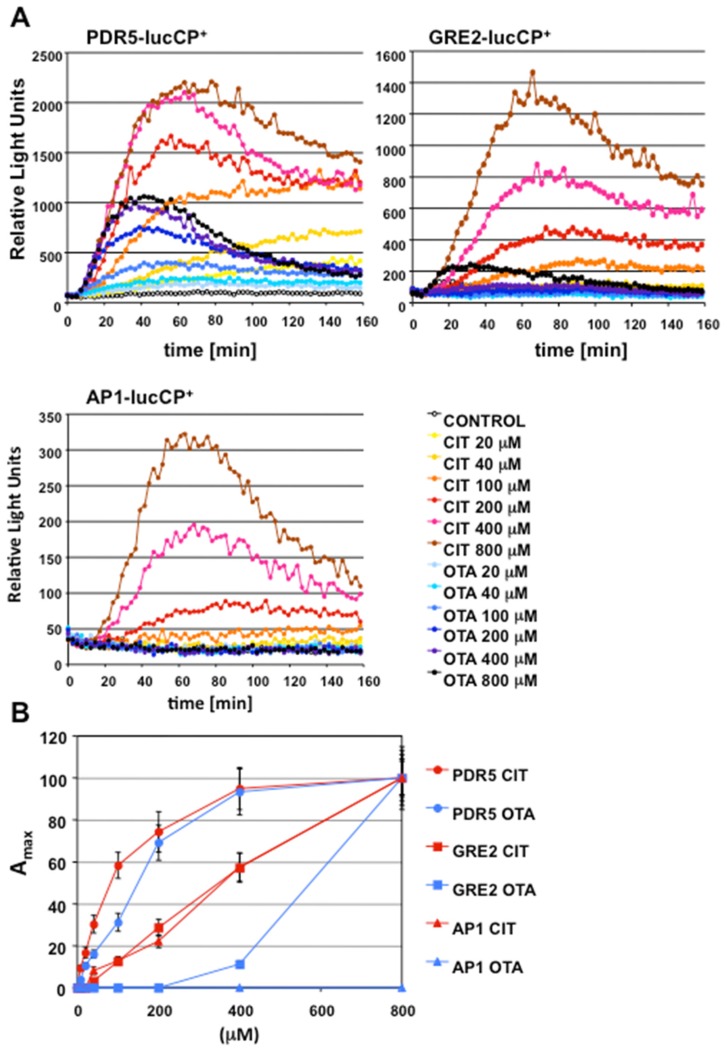
CIT, as opposed to OTA, induces a sensitive oxidative and general stress response in yeast cells. (**A**) OTA and CIT induction of the PDR5–, GRE2– and AP1–luciferase reporters. Live cell reporter fusions with destabilized luciferase were used in yeast wild type cells and the induction of both genes was measured in real time upon the indicated mycotoxin doses. The data are derived from three independent culture aliquots and had an error of <15%. (**B**) Dose-response profiles of the different luciferase reporters. The maximal steady-state activity (A_max_) was calculated for each reporter strain and toxin dose and plotted against the mycotoxin concentration. A_max_ for the highest toxin exposure was arbitrarily set to 100.

We next wanted to test whether CIT and OTA caused different biological effects in the first instances of exposure. We therefore applied different live cell gene expression reporters in yeast cells to monitor transcriptional responses, which are triggered by distinct biological stimuli. Since we have previously shown that the Pdr5 drug transporter is important for the response to both CIT and OTA, we used a *PDR5*–luciferase expressing strain to monitor the induction of *PDR5*, which is activated by the accumulation of both toxins in the cell interior and not linked to a specific type of stress. Furthermore, we recorded the activation of two additional reporters, the general stress-inducible GRE2–luciferase, and the oxidative stress-inducible AP1–luciferase fusion [42]. We obtained the complete dose-response profiles of all three reporter strains upon increasing CIT and OTA exposures (Figure 4A). The relationship between the toxin dose and the transcriptional output (A_max_) allowed us to visualize the relative sensibilities, with which each reporter was activated by the two mycotoxins (Figure 4B), and to observe important differences. Both CIT and OTA induced the PDR5–lucCP reporter with similar dose-response kinetics. However, the stress-specific GRE2 and AP1 reporters were activated by CIT in a much more sensitive manner as compared to OTA (Figure 4B). Remarkably, the oxidative stress specific AP1–luciferase reporter remained completely uninduced even at the highest OTA concentrations. These data, together with the previous phenotypic analysis of specific yeast mutants, clearly indicated that CIT and OTA have divergent biological effects in cells. Taking together all the results presented here, CIT exposure causes strong oxidative stress, which triggers a massive transcriptional antioxidant and drug extrusion response, while OTA mainly deregulates developmental genes and only marginally induces the antioxidant defense.

## 3. Discussion

Here we compare the toxicity targets of the mycotoxins ochratoxin A and citrinin using yeast as a model. *Saccharomyces cerevisiae* is a very suitable organism to investigate the adaptive response triggered by OTA and CIT, because both toxins cause rapid and profound changes in gene expression in yeast. Moreover, yeast transcriptional responses can be compared quantitatively in real time for different stress-specific reporters and additionally on a genomic scale. These approaches are thus suitable as a diagnostic tool to discern divergent and common biological effects of toxins. It is important to note that yeast cells seem to resist much higher CIT and OTA doses as compared to mammalian cells. The reasons for this might be a very efficient extrusion by multidrug transporters in this organism—which is shown here as being especially relevant for CIT detoxification—or the function of the yeast cell wall, which might serve as a primary barrier for mycotoxins. The adsorption by the yeast cell wall is actually an emerging biotechnological approach to control the concentration of different mycotoxins including OTA [43,44].

A common defense strategy of eukaryotic cells against many unrelated toxic compounds and xenobiotics is the activation of multidrug transporters at the plasma membrane [45,46]. In yeast cells, such as in other fungi and human cells, the intracellular levels of toxic molecules are directly sensed by specialized transcription factors, which in turn activate the expression of multidrug transporter genes in an attempt to physically extrude the toxic agents from the cell interior [47]. Here we take advantage of a specific drug efflux pump, Pdr5, which seems to be important for both CIT and OTA detoxification. Mutants for Pdr5 respond in a much more sensitive manner to both mycotoxins, as indicated by a more pronounced transcriptional activation of stress reporters by lower toxin concentrations. Although not tested directly, we assume that *pdr5* mutant cells accumulate higher CIT and OTA concentrations. We took advantage of this sensitivity phenotype to carry out genomic profiling experiments. The use of a hypersensitive mutant strain and the selection of optimized toxin concentrations and time points for sample preparation favored the identification of many significantly deregulated gene functions in the immediate response to both compounds. We show that the expression of the *PDR5* gene is activated by CIT and OTA with similar dose response profiles (Figure 3B). This result indicates that both mycotoxins are similarly taken up by yeast cells and that the differences in the gene expression profiles are not due to a differential intracellular accumulation of the two compounds.

Citrinin induces the expression of many different multidrug transporters, and the functional category “Drug membrane transport” is significantly enriched among the CIT target genes. Seven multidrug exporter genes are highly induced by CIT: *FLR1*, *ATR1*, *SNQ2*, *PDR15*, *PDR10*, *PDR16*, and *YOR1*. All of these transporters are localized, at least in part, at the plasma membrane. Thus the inducible active transport of CIT from the cytosol to the cell exterior is an important feature of detoxification of this mycotoxin in yeast cells. Accordingly, we detect an increased sensitivity to CIT by the loss of individual transporters such as Pdr5, Snq2 or Yor1. OTA, however, has a much weaker impact on the induction of the multidrug extrusion system, which coincides with the CIT response only in the moderate induction of the *SNQ2* gene. Of note, the yeast pleiotropic drug response is activated by the mere presence of the compound in the cell interior and also by the cytotoxic stress triggered by the compound. Thus the higher impact of CIT on the ROS balance of the cell as compared to OTA could result in a much more profound transcriptional activation of the multidrug export system.

Here we show that the predominant mechanism of CIT toxicity is the induction of oxidative stress. Moreover, oxidative stress reporters are immediately upregulated upon CIT exposure and yeast mutants with a weakened antioxidant defense are hypersensitive to this mycotoxin, which altogether suggests that the induction of ROS inside cells is a primary mode of CIT action. Our result is in agreement with a previous transcriptomic assay in yeast upon prolonged CIT treatment [41] and with several studies showing CIT induced oxidative damage in diverse cellular models from yeast to humans [26,27,28,30]. As a consequence, external addition of antioxidants usually alleviates CIT toxicity [25,48,49]. How, at the molecular level, CIT increases intracellular ROS levels is currently unknown, however, several studies have implied an inhibition of mitochondrial respiration in CIT-activated oxidative stress [29,31,50]. On the other hand, we demonstrate here that OTA has a much less pronounced impact on the yeast antioxidant response at the genomic level, which is further corroborated by specific oxidative stress reporters. Thus, oxidative stress might not be the primary toxicity mechanism for this mycotoxin. This divergent impact of CIT and OTA on ROS production is in complete agreement with a recent study showing that CIT-, but not OTA-induced hepatotoxicity, is efficiently counteracted by antioxidant treatment [49]. However, the genomic response of yeast to OTA does include the upregulation of some antioxidant functions, which interestingly are different from the antioxidant genes induced by CIT. OTA induces, for example, the expression of both mitochondrial/peroxisomal and cytosolic catalases (Ctt1 and Cta1), while CIT preferentially stimulates enzymatic functions involved in glutathione metabolism (Ecm4, Glr1, Gsh1, Gtt2, and Grx2). Thus, apart from considerable differences in absolute ROS induction, it might be possible that CIT and OTA produce distinct types of reactive oxygen species. These differences are striking because CIT and OTA are structurally related mycotoxins. Both share a dihydroisocoumarin moiety as the central structure element, which is coupled to the amino acid phenylalanine in the case of OTA. However, a functional divergence has been suggested also with respect to the environmental conditions, which induce the biosynthesis of CIT or OTA in their natural producer *Penicillium verrucosum*. Here different stress conditions, such as oxidative or salt stress, have been shown to differentially favor the production of one mycotoxin over the other [19,20].

Despite a large scientific effort, the critical mechanism underlying OTA cytotoxicity still remains unknown. Oxidative stress has been widely implied in OTA action [15], but it certainly cannot explain the carcinogenic properties of this mycotoxin. Here we confirm that OTA is able to trigger an antioxidant response in yeast, however, ROS production is not the principle effect of OTA. This is in agreement with recent studies, which demonstrate in rats that renal carcinogenicity and cell cycle aberrations caused by OTA cannot be explained by oxidative damage [51,52]. Here we show that OTA treatment causes a general deregulation of developmental genes in yeast. This effect is OTA-specific and is not observed upon CIT exposure. The affected gene functions are related to the processes of meiosis and sporulation, which are normally tightly repressed in haploid yeast cells such as the strains used here for the transcriptomic experiments. Therefore, OTA seems to cause a genomic reprogramming of a developmental process, which is normally exclusively triggered in diploid yeast cells upon the appropriate environmental stimuli [53,54]. A tight epigenetic control, composed of specific DNA-binding factors which recruit histone deacetylases such as the Hst1 sirtuin to meiotic and sporulation genes, are known in yeast to assure repression of these developmental genes in haploid cells [55,56,57]. How OTA can interfere with the epigenetic control of silenced genes in yeast is currently only speculative, but opens an emerging research towards the biological function of this mycotoxin. This is of outstanding importance because the interference with gene silencing and the function of sirtuin histone deacetylases are hallmarks in the reprogramming of cancer cells [58,59] and thus could provide insights into the carcinogenic function of OTA. Taken together, our results demonstrate divergent biological effects of two related mycotoxins, which will be important for understanding their toxicity mechanisms at the molecular level.

## 4. Materials and Methods

### 4.1. Yeast Strains and Growth Conditions

*Saccharomyces cerevisiae* strains used in this study were: wild type BY4741 (*MATa*; *his3*Δ*1*; *leu2*Δ*0*; *met15*Δ*0*; *ura3*Δ*0*) and the mutant alleles *yap1::KanMX4*; *skn7::KanMX4*; *yor1::KanMX4*; *pdr5::KanMX4*; *snq2::KanMX4*. For luciferase assays the cells were transformed with the respective lucCP^+^ fusion plasmids and grown in synthetic dextrose (SD) medium which contained 0.67% yeast nitrogen base, 50 mM succinic acid pH 5.5, 2% dextrose, 100 mg/L methionine, 100 mg/L leucine, and 25 mg/L uracil. For CIT and OTA sensitivity assays on agar plates, the respective yeast strains were grown in SD liquid medium containing 2% dextrose to exponential growth phase and then incubated with 400 μM of CIT or OTA for the indicated time in small culture aliquots in multiwell plates at 28 °C. Citrinin and ochratoxin A were purchased from Enzo Life Sciences (Farmingdale, NY, USA), and stock solutions were prepared with DMSO as the solvent.

### 4.2. Plasmid Constructions

The destabilized luciferase reporter fusions with the natural *GRE2* or *SOD2* promoters are described elsewhere [60,61]. Briefly, the *GRE2*–lucCP^+^ fusion contains the upstream 940 nucleotides of the *GRE2* gene fused with the destabilized luciferase lucCP^+^ gene in a centromeric *HIS3*-containing yeast expression plasmid. The *SOD2*–lucCP^+^ fusion contains the upstream 977 nucleotides of the *SOD2* gene in the same vector backbone. The AP-1-specific destabilized luciferase reporter is described in [60]. Briefly, it contains a triple insertion of the AP-1 promoter element in the *CYC1* core promoter fused to lucCP^+^ in centromeric *HIS3*-containing yeast expression plasmids. A PDR5–luciferase expressing reporter strain was created by integrative transformation of a PDR5–lucCP^+^–Kan MX DNA cassette into yeast wild type strain BY4741 to replace the endogenous *PDR5* gene with the destabilized luciferase gene.

### 4.3. Live Cell Luciferase Assays

Yeast strains transformed with the respective luciferase reporter plasmids were grown at 28 °C overnight in SD medium to OD = 2 at 600 nm. The culture volume necessary for the entire luciferase assay was incubated on a roller at 28 °C for 90 min with 0.5 mM luciferin (Synchem, Felsberg, Germany) from a 10 mM stock solution in Dimethylsulfoxide. The culture was then distributed in 120 μL aliquots in white 96-well plates (Nunc, Penfield, NY, USA) and growing concentrations of CIT or OTA were added from a stock solution in DMSO. In Figure 1, 200 μM (= 50 ppm), 800 μM (= 200 ppm), and 1600 μM (= 400 ppm) of CIT and 124 μM (= 50 ppm), 497 μM (= 200 ppm), and 994 μM (= 400 ppm) of OTA were applied. Additionally, a constant dose of 200 μM (= 50 ppm) of CIT was combined with growing OTA concentrations (124 μM (= 50 ppm), 497 μM (= 200 ppm), and 994 μM (= 400 ppm)). In Figure 3, 20 μM, 40 μM, 100 μM, 200 μM, 400 μM, and 800 μM of CIT or OTA were used. The mock-treated samples contained the same concentration of solvent without the mycotoxin. The light emission from the culture aliquots was continuously recorded in a GloMax Multidetection System (Promega, Madison, WI, USA) in the luminometer mode. Data were normalized for the absolute number of cells used in the assay and processed in Microsoft Excel (2010). For each condition, three independent culture aliquots were analyzed. The maximal luciferase activity depicted in Figure 1C and Figure 4B was calculated by correcting the maximal light emission for each treatment with the value obtained for the mock-treated culture.

### 4.4. Yeast Sensitivity Assays

For plate assays, the yeast strains under study were grown in SD liquid medium to exponential growth phase. 1:1, 1:10 and 1:100 dilutions of culture aliquots were then distributed in multiwell plates and exposed for the indicated time to CIT or OTA added from stock solutions in DMSO. Equal amounts of cells were then plated on fresh yeast extract peptone dextrose (YPD) agar plates, which were incubated at 28 °C for 2 days.

### 4.5. Microarray Experiments and Analysis

For the comparison of the transcriptome upon various mycotoxin treatments, the *pdr5* mutant strain was used. Cells were grown in SD medium until exponential phase and then subjected to four different toxin treatments: control (mock treated with solvent), CIT (200 ppm for 60 min), OTA (200 ppm for 30 min), and a combination of both mycotoxins CIT/OTA (100 ppm each for 30 min). Total RNA was prepared from four independent culture aliquots for each condition using the acid phenol extraction method. Total RNA was further purified with the RNeasy Mini kit (Qiagen, Valencia, CA, USA). The samples were labeled using the one-color method with Cy3 fluorophore, hybridized to Agilent Yeast Gene Expression 8 × 15 K microarrays, and scanned with Agilent DNA Microarray Scanner (G2505B, Agilent Technologies, Santa Clara, CA, USA). Raw data were obtained using the Feature Extraction software 9.5.1 (Agilent Technologies, Santa Clara, CA, USA, 2007). These procedures were performed by the Genomic Service of the Instituto de Biología Molecular y Celular de Plantas (IBMCP, Valencia, Spain). Data analysis was performed using GeneSpring 12.6 (Agilent Technologies, Santa Clara, CA, USA). Data were normalized using the quantile method and then statistically analyzed with the Student *t*-Test. Significant differences in gene expression were selected using a *p*-value < 0.05. To avoid the detection of false positives, a multiple testing correction (Bonferroni FWER) was applied to obtain corrected *p*-values. The complete dataset from all transcriptomic experiments of this publication has been assigned accession number GSE84187 in the Gene Expression Omnibus (GEO) Database. Significantly enriched functional gene groups were identified with the YeastMine Gene Ontology (GO) search option of the *Saccharomyces cerevisiae* Genome Database (SGD).

## Figures and Tables

**Table 1:** Genes > 5-fold upregulated upon CIT (citrinin) exposure.

Gene	Standard Name	FC *	*p*-Value	Description
YPL171C	*OYE3*	473.1	3.00 × 10^−8^	Conserved NADPH oxidoreductase containing flavin mononucleotide (FMN)
YFL056C	*AAD6*	252.4	9.60 × 10^−7^	Putative aryl-alcohol dehydrogenase
YDL243C	*AAD4*	252.1	1.77 × 10^−9^	Putative aryl-alcohol dehydrogenase
YCL026C-A	*FRM2*	177.2	1.77 × 10^−5^	Type II nitroreductase
YLL060C	*GTT2*	142.4	4.50 × 10^−5^	Glutathione S-transferase
YBR008C	*FLR1*	120.6	1.83 × 10^−7^	Plasma membrane multidrug transporter of the major facilitator superfamily
YCL026C-B	*HBN1*	61.7	1.81 × 10^−6^	Protein of unknown function
YGR213C	*RTA1*	57.8	1.77 × 10^−8^	Protein involved in 7-aminocholesterol resistance
YML116W	*ATR1*	54.8	1.45 × 10^−7^	Multidrug efflux pump of the major facilitator superfamily
YKR076W	*ECM4*	51.6	6.10 × 10^−6^	Omega class glutathione transferase
YML131W	*-*	41.2	9.44 × 10^−3^	Protein of unknown function
YHR139C	*SPS100*	35.2	4.78 × 10^−7^	Protein required for spore wall maturation
YFL057C	*AAD16*	33.5	2.12 × 10^−2^	Putative aryl-alcohol dehydrogenase
YDR011W	*SNQ2*	31.1	6.78 × 10^−7^	Plasma membrane ATP-binding cassette (ABC) transporter
YOL151W	*GRE2*	25.8	2.28 × 10^−6^	3-methylbutanal reductase and NADPH-dependent methylglyoxal reductase
YKL086W	*SRX1*	25.6	5.22 × 10^−7^	Sulfiredoxin
YDR406W	*PDR15*	18.1	1.12 × 10^−6^	Plasma membrane ATP binding cassette (ABC) transporter
YLR108C	*-*	16.6	5.77 × 10^−8^	Protein of unknown function
YDL020C	*RPN4*	15.8	9.41 × 10^−8^	Transcription factor that stimulates expression of proteasome genes
YNL117W	*MLS1*	15.1	3.95 × 10^−6^	Malate synthase
YOR328W	*PDR10*	14.5	2.41 × 10^−8^	ATP-binding cassette (ABC) transporter
YHR199C	*AIM46*	13.3	1.58 × 10^−7^	Putative protein of unknown function
YHR029C	*YHI9*	12.9	3.86 × 10^−7^	Protein of unknown function
YGR256W	*GND2*	10.9	5.58 × 10^−7^	6-phosphogluconate dehydrogenase
YBR244W	*GPX2*	10.5	3.56 × 10^−6^	Phospholipid hydroperoxide glutathione peroxidase
YFL030W	*AGX1*	10.3	4.28 × 10^−8^	Alanine:glyoxylate aminotransferase (AGT)
YDR453C	*TSA2*	9.6	2.01 × 10^−7^	Stress inducible cytoplasmic thioredoxin peroxidase
YER143W	*DDI1*	9.5	8.66 × 10^−5^	DNA damage-inducible v-SNARE binding protein
YNR074C	*AIF1*	9.1	4.46 × 10^−7^	Mitochondrial cell death effector
YER042W	*MXR1*	9.0	2.11 × 10^−6^	Methionine-S-sulfoxide reductase
YJL101C	*GSH1*	8.9	1.30 × 10^−7^	Gamma glutamylcysteine synthetase
YHR138C	*-*	8.8	1.42 × 10^−3^	Protein of unknown function
YHL036W	*MUP3*	8.6	1.13 × 10^−5^	Low affinity methionine permease
YNL129W	*NRK1*	8.5	1.61 × 10^−5^	Nicotinamide riboside kinase
YPR200C	*ARR2*	8.1	1.57 × 10^−4^	Arsenate reductase
YER103W	*SSA4*	7.8	2.65 × 10^−5^	Heat shock protein
YJL045W	*-*	7.7	3.32 × 10^−7^	Minor succinate dehydrogenase isozyme
YPL027W	*SMA1*	7.7	9.86 × 10^−7^	Protein of unknown function involved in prospore membrane assembly
YGR010W	*NMA2*	7.5	1.02 × 10^−7^	Nicotinic acid mononucleotide adenylyltransferase
YMR169C	*ALD3*	7.4	6.10 × 10^−4^	Cytoplasmic aldehyde dehydrogenase
YDR132C	*-*	7.3	1.74 × 10^−6^	Protein of unknown function
YOR162C	*YRR1*	7.2	1.24 × 10^−7^	Zn_2_-Cys_6_ zinc-finger transcription factor
YMR038C	*CCS1*	6.9	6.96 × 10^−5^	Copper chaperone for superoxide dismutase Sod1p
YJL219W	*HXT9*	6.9	1.67 × 10^−7^	Putative hexose transporter
YER142C	*MAG1*	6.8	5.46 × 10^−7^	3-methyl-adenine DNA glycosylase
YBR046C	*ZTA1*	6.7	1.13 × 10^−5^	NADPH-dependent quinone reductase
YNL231C	*PDR16*	6.6	7.41 × 10^−3^	Phosphatidylinositol transfer protein (PITP)
YPL091W	*GLR1*	6.5	1.49 × 10^−5^	Cytosolic and mitochondrial glutathione oxidoreductase
YGR281W	*YOR1*	6.4	2.16 × 10^−3^	Plasma membrane ATP-binding cassette (ABC) transporter
YGR197C	*SNG1*	6.3	3.47 × 10^−7^	Protein involved in resistance to nitrosoguanidine and 6-azauracil
YNL155W	*CUZ1*	6.1	5.38 × 10^−3^	Protein with a role in the ubiquitin-proteasome pathway
YAL054C	*ACS1*	6.1	3.74 × 10^−7^	Acetyl-coA synthetase isoform
YOL119C	*MCH4*	6.1	1.27 × 10^−5^	Protein with similarity to mammalian monocarboxylate permeases
YDL168W	*SFA1*	6.0	1.21 × 10^−5^	Bifunctional alcohol dehydrogenase and formaldehyde dehydrogenase
YCR021C	*HSP30*	6.0	5.37 × 10^−3^	Negative regulator of the H(+)-ATPase Pma1p
YBR256C	*RIB5*	5.9	1.15 × 10^−3^	Riboflavin synthase
YOR052C	*TMC1*	5.8	9.56 × 10^−3^	AN1-type zinc finger protein of unknown function
YOL155C	*HPF1*	5.8	6.09 × 10^−5^	Haze-protective mannoprotein
YMR318C	*ADH6*	5.8	7.64 × 10^−3^	NADPH-dependent medium chain alcohol dehydrogenase
YJL082W	*IML2*	5.8	4.56 × 10^−4^	Protein of unknown function
YKL051W	*SFK1*	5.6	6.62 × 10^−6^	Plasma membrane protein that may act to generate normal levels of PI4P
YER185W	*PUG1*	5.6	3.14 × 10^−5^	Plasma membrane protein involved in protoprophyrin and heme transport
YIR017C	*MET28*	5.6	3.48 × 10^−6^	Basic leucine zipper (bZIP) transcriptional activator in the Cbf1p-Met4p-Met28p complex
YHL024W	*RIM4*	5.5	4.66 × 10^−6^	Putative RNA-binding protein
YGR243W	*MPC3*	5.4	7.07 × 10^−5^	Highly conserved subunit of mitochondrial pyruvate carrier
YGL010W	*MPO1*	5.3	7.58 × 10^−6^	Protein involved in metabolism of phytosphingosine
YDR513W	*GRX2*	5.1	6.09 × 10^−3^	Cytoplasmic glutaredoxin
YHR179W	*OYE2*	5.1	1.04 × 10^−2^	Conserved NADPH oxidoreductase containing flavin mononucleotide (FMN)
YDR059C	*UBC5*	5.1	2.39 × 10^−4^	Ubiquitin-conjugating enzyme
YMR276W	*DSK2*	5.0	5.01 × 10^−3^	Nuclear-enriched ubiquitin-like polyubiquitin-binding protein

* Fold change (FC) refers to the fold induction of the genes as compared to the untreated control.

**Table 2:** Genes > 5-fold upregulated upon OTA (ochratoxin A) exposure.

Gene	Standard Name	FC *	*p*-Value	Description
YER106W	*MAM1*	60.2	2.77 × 10^−8^	Monopolin
YGR225W	*AMA1*	57.4	9.19 × 10^−10^	Activator of meiotic anaphase promoting complex (APC/C)
YER179W	*DMC1*	40.5	5.34 × 10^−7^	Meiosis-specific recombinase
YOR298W	*MUM3*	33.5	9.62 × 10^−4^	Protein of unknown function
YFL011W	*HXT10*	33.2	1.38 × 10^−7^	Putative hexose transporter
YLL046C	*RNP1*	27.3	1.08 × 10^−7^	Ribonucleoprotein
YER104W	*RTT105*	26.0	3.22 × 10^−8^	Protein with a role in regulation of Ty1 transposition
YLR377C	*FBP1*	23.3	1.62 × 10^−7^	Fructose-1,6-bisphosphatase
YDR523C	*SPS1*	22.7	6.27 × 10^−6^	Putative protein serine/threonine kinase
YHR176W	*FMO1*	20.2	1.11 × 10^−5^	Flavin-containing monooxygenase
YBR040W	*FIG1*	19.7	1.16 × 10^−7^	Integral membrane protein
YGR059W	*SPR3*	18.6	4.74 × 10^−5^	septin protein involved in sporulation
YEL039C	*CYC7*	16.9	6.54 × 10^−7^	Cytochrome c isoform 2
YMR101C	*SRT1*	16.7	3.73 × 10^−7^	Forms the dehydrodolichyl diphosphate syntase (DDS) complex with NUS1
YDR218C	*SPR28*	14.1	1.11 × 10^−6^	Meiotic septin
YDR256C	*CTA1*	13.5	7.51 × 10^−8^	Catalase A
YIL113W	*SDP1*	13.3	2.62 × 10^−7^	Stress-inducible dual-specificity MAP kinase phosphatase
YOL123W	*HRP1*	12.9	1.98 × 10^−6^	Subunit of cleavage factor I complex
YGL254W	*FZF1*	12.6	2.03 × 10^−7^	Transcription factor involved in sulfite metabolism
YPL201C	*YIG1*	12.4	3.23 × 10^−5^	Protein that interacts with glycerol 3-phosphatase
Q0275	*COX3*	12.3	1.01 × 10^−4^	Subunit III of cytochrome c oxidase (Complex IV)
YFL055W	*AGP3*	12.3	2.34 × 10^−6^	Low-affinity amino acid permease
YDR259C	*YAP6*	11.4	1.88 × 10^−5^	Basic leucine zipper (bZIP) transcription factor
YPR193C	*HPA2*	11.3	2.74 × 10^−5^	Tetrameric histone acetyltransferase
YOR378W	*AMF1*	11.3	2.33 × 10^−6^	Low affinity NH4+ transporter
YLL042C	*ATG10*	11.3	3.47 × 10^−6^	Conserved E2-like conjugating enzyme
YIL101C	*XBP1*	11.1	3.43 × 10^−4^	Transcriptional repressor
YBR018C	*GAL7*	11.0	2.12 × 10^−5^	Galactose-1-phosphate uridyl transferase
YEL019C	*MMS21*	10.9	6.11 × 10^−6^	SUMO ligase and component of the SMC5-SMC6 complex
YPR040W	*TIP41*	10.9	3.19 × 10^−5^	Protein that interacts with Tap42p
YPL033C	*SRL4*	10.7	1.75 × 10^−6^	Protein of unknown function
YLL057C	*JLP1*	10.5	1.82 × 10^−6^	Fe(II)-dependent sulfonate/alpha-ketoglutarate dioxygenase
YGR142W	*BTN2*	10.3	2.34 × 10^−5^	v-SNARE binding protein
YPL279C	*FEX2*	10.3	2.64 × 10^−7^	Protein involved in fluoride export
YHL022C	*SPO11*	10.2	2.70 × 10^−7^	Meiosis-specific protein
YKL055C	*OAR1*	10.0	2.10 × 10^−6^	Mitochondrial 3-oxoacyl-[acyl-carrier-protein] reductase
YNL009W	*IDP3*	10.0	1.42 × 10^−2^	Peroxisomal NADP-dependent isocitrate dehydrogenase
YOR297C	*TIM18*	9.9	3.75 × 10^−5^	Component of the mitochondrial TIM22 complex
YER053C-A	*-*	9.8	7.45 × 10^−6^	Protein of unknown function
YPL027W	*SMA1*	9.7	1.50 × 10^−7^	Protein of unknown function
YBR074W	*PFF1*	9.6	5.70 × 10^−6^	Multi-spanning vacuolar membrane protease
YEL048C	*TCA17*	9.6	2.14 × 10^−7^	Component of transport protein particle (TRAPP) complex II
YGR197C	*SNG1*	9.2	7.32 × 10^−8^	Protein involved in resistance to nitrosoguanidine and 6-azauracil
YJR047C	*ANB1*	9.2	1.29 × 10^−6^	Translation elongation factor eIF-5A
YKL093W	*MBR1*	9.0	3.41 × 10^−5^	Protein involved in mitochondrial functions and stress response
YGR212W	*SLI1*	9.0	2.03 × 10^−5^	*N*-acetyltransferase
YCL026C-A	*FRM2*	8.8	1.66 × 10^−6^	Type II nitroreductase
YEL072W	*RMD6*	8.7	6.39 × 10^−7^	Protein required for sporulation
YML054C	*CYB2*	8.5	2.74 × 10^−6^	Cytochrome b2 (l-lactate cytochrome-c oxidoreductase)
YNL187W	*SWT21*	8.5	6.08 × 10^−6^	Protein involved in mRNA splicing
YNR064C	*-*	8.5	1.99 × 10^−5^	Epoxide hydrolase
YBR065C	*ECM2*	8.4	9.49 × 10^−6^	Pre-mRNA splicing factor
YPL171C	*OYE3*	8.4	6.43 × 10^−6^	Conserved NADPH oxidoreductase containing flavin mononucleotide (FMN)
YGL212W	*VAM7*	8.4	1.02 × 10^−4^	Vacuolar SNARE protein
YOR390W	*FEX1*	8.2	3.59 × 10^−6^	Protein involved in fluoride export
YMR069W	*NAT4*	8.1	1.76 × 10^−4^	*N*-alpha-acetyl-transferase
YDL020C	*RPN4*	8.0	3.51 × 10^−7^	Transcription factor that stimulates expression of proteasome genes
YDR171W	*HSP42*	8.0	6.87 × 10^−6^	Small heat shock protein (sHSP) with chaperone activity
YER054C	*GIP2*	7.9	2.59 × 10^−6^	Putative regulatory subunit of protein phosphatase Glc7p
YPR151C	*SUE1*	7.9	9.84 × 10^−7^	Protein required for degradation of unstable forms of cytochrome c
YGR131W	*FHN1*	7.7	1.62 × 10^−6^	Protein of unknown function
YEL061C	*CIN8*	7.6	1.15 × 10^−5^	Kinesin motor protein
YDR079W	*PET100*	7.6	4.29 × 10^−6^	Chaperone that specifically facilitates the assembly of cytochrome c oxidase
YKL051W	*SFK1*	7.6	1.38 × 10^−4^	Plasma membrane protein
YMR017W	*SPO20*	7.5	1.72 × 10^−3^	Meiosis-specific subunit of the t-SNARE complex
YDR011W	*SNQ2*	7.5	4.53 × 10^−7^	Plasma membrane ATP-binding cassette (ABC) transporter
YOR152C	*ATG40*	7.4	4.01 × 10^−5^	Autophagy receptor
YLR312C	*ATG39*	7.4	2.53 × 10^−7^	Autophagy receptor
YBL078C	*ATG8*	7.3	7.40 × 10^−7^	Component of autophagosomes and Cvt vesicles
YPL186C	*UIP4*	7.2	4.47 × 10^−4^	Protein that interacts with Ulp1p
YLR142W	*PUT1*	7.1	2.11 × 10^−6^	Proline oxidase
YOR065W	*CYT1*	7.0	4.71 × 10^−5^	Cytochrome c1
YOL149W	*DCP1*	7.0	1.35 × 10^−3^	Subunit of the Dcp1p-Dcp2p decapping enzyme complex
Q0250	*COX2*	6.7	3.78 × 10^−2^	Subunit II of cytochrome c oxidase (Complex IV)
YDR402C	*DIT2*	6.6	1.08 × 10^−3^	*N*-formyltyrosine oxidase
YGR243W	*MPC3*	6.6	1.70 × 10^−5^	Highly conserved subunit of the mitochondrial pyruvate carrier (MPC)
YOR005C	*DNL4*	6.6	5.57 × 10^−6^	DNA ligase
YJR010W	*MET3*	6.6	9.83 × 10^−7^	ATP sulfurylase
YLR151C	*PCD1*	6.5	2.79 × 10^−6^	8-oxo-dGTP diphosphatase
YNL158W	*PGA1*	6.3	4.04 × 10^−4^	Essential component of GPI-mannosyltransferase II
YDR524C	*AGE1*	6.3	8.02 × 10^−7^	ADP-ribosylation factor (ARF) GTPase activating protein (GAP) effector
YNL012W	*SPO1*	6.3	4.68 × 10^−6^	Meiosis-specific prospore protein
YGL240W	*DOC1*	6.3	6.44 × 10^−5^	Processivity factor
YDR076W	*RAD55*	6.3	1.32 × 10^−4^	Protein that stimulates strand exchange
YOR192C	*THI72*	6.3	7.85 × 10^−6^	Transporter of thiamine or related compound
YMR251W	*GTO3*	6.3	2.35 × 10^−5^	Omega class glutathione transferase
YDR185C	*UPS3*	6.2	4.77 × 10^−6^	Mitochondrial protein of unknown function
YNL014W	*HEF3*	6.2	1.32 × 10^−4^	Translational elongation factor EF-3
YML087C	*AIM33*	6.2	1.01 × 10^−4^	Putative protein of unknown function
YNR034W	*SOL1*	6.2	7.19 × 10^−7^	Protein with a possible role in tRNA export
YDR070C	*FMP16*	6.1	3.24 × 10^−4^	Protein of unknown function
YJR129C	*EFM3*	6.1	4.06 × 10^−2^	S-adenosylmethionine-dependent methyltransferase
Q0045	*COX1*	6.0	1.76 × 10^−2^	Subunit I of cytochrome c oxidase (Complex IV)
YNL036W	*NCE103*	5.9	4.88 × 10^−5^	Carbonic anhydrase
YOR178C	*GAC1*	5.9	6.08 × 10^−4^	Regulatory subunit for Glc7p type-1 protein phosphatase (PP1)
YGR088W	*CTT1*	5.9	8.13 × 10^−5^	Cytosolic catalase T
YDL247W	*MPH2*	5.8	2.28 × 10^−5^	Alpha-glucoside permease
YCL066W	*HMLALPHA1*	5.7	6.90 × 10^−4^	Silenced copy of ALPHA1 at HML
YNL077W	*APJ1*	5.6	3.33 × 10^−6^	Chaperone with a role in SUMO-mediated protein degradation
YKL095W	*YJU2*	5.6	1.29 × 10^−3^	Essential protein required for pre-mRNA splicing
YJL030W	*MAD2*	5.6	1.64 × 10^−4^	Component of the spindle-assembly checkpoint complex
YHL016C	*DUR3*	5.6	9.87 × 10^−7^	Plasma membrane transporter for urea and polyamines
YNL188W	*KAR1*	5.6	1.64 × 10^−4^	Protein involved in karyogamy and spindle pole body duplication
YGR234W	*YHB1*	5.6	1.02 × 10^−5^	Nitric oxide oxidoreductase
YCR040W	*MATALPHA1*	5.5	6.76 × 10^−4^	Transcriptional co-activator that regulates mating-type-specific genes
YFL016C	*MDJ1*	5.5	2.05 × 10^−4^	Co-chaperone that stimulates HSP70 protein Ssc1p ATPase activity
YNL194C	*-*	5.4	4.89 × 10^−4^	Integral membrane protein
YDR475C	*JIP4*	5.3	2.01 × 10^−3^	Protein of unknown function
YJR160C	*MPH3*	5.3	8.87 × 10^−5^	Alpha-glucoside permease
YCR104W	*PAU3*	5.3	1.92 × 10^−3^	Member of the seripauperin multigene family
YIL084C	*SDS3*	5.3	6.30 × 10^−6^	Component of the Rpd3L histone deacetylase complex
YIL056W	*VHR1*	5.1	3.53 × 10^−3^	Transcriptional activator
YAR020C	*PAU7*	5.0	1.56 × 10^−4^	Member of the seripauperin multigene family
YDR227W	*SIR4*	5.0	1.71 × 10^−5^	Silent information regulator
YLR376C	*PSY3*	5.0	6.70 × 10^−6^	Component of Shu complex (aka PCSS complex)

* Fold change (FC) refers to the fold induction of the genes as compared to the untreated control.

**Table 3:** Genes > 5-fold upregulated upon the combined CIT/OTA exposure.

Gene	Standard Name	FC *	*p*-Value	Description
YPL171C	*OYE3*	199.6	1.29 × 10^−4^	Conserved NADPH oxidoreductase containing flavin mononucleotide (FMN)
YDL243C	*AAD4*	46.5	1.49 × 10^−9^	Putative aryl-alcohol dehydrogenase
YFL056C	*AAD6*	41.2	1.16 × 10^−7^	Putative aryl-alcohol dehydrogenase
YLL060C	*GTT2*	34.6	1.44 × 10^−9^	Glutathione S-transferase capable of homodimerization
YBR008C	*FLR1*	28.0	1.21 × 10^−7^	Plasma membrane transporter of the major facilitator superfamily
YML131W	*-*	24.2	2.41 × 10^−6^	Protein of unknown function
YOL151W	*GRE2*	21.9	1.17 × 10^−4^	3-methylbutanal reductase and NADPH-dependent methylglyoxal reductase
YCL026C-A	*FRM2*	21.5	1.53 × 10^−8^	Type II nitroreductase
YMR101C	*SRT1*	21.3	2.64 × 10^−8^	Forms the dehydrodolichyl diphosphate syntase (DDS) complex with NUS1
YGR225W	*AMA1*	20.5	3.87 × 10^−7^	Activator of meiotic anaphase promoting complex (APC/C)
YDL020C	*RPN4*	19.5	4.24 × 10^−4^	Transcription factor that stimulates expression of proteasome genes
YDR256C	*CTA1*	18.8	4.53 × 10^−9^	Catalase A
YGR197C	*SNG1*	18.7	5.35 × 10^−9^	Protein involved in resistance to nitrosoguanidine and 6-azauracil
YKL051W	*SFK1*	18.7	6.62 × 10^−8^	Plasma membrane protein that may act to generate normal levels of PI4P
YML116W	*ATR1*	16.3	9.44 × 10^−6^	Multidrug efflux pump of the major facilitator superfamily
YGR142W	*BTN2*	15.2	7.12 × 10^−6^	v-SNARE binding protein
YHR087W	*RTC3*	15.0	1.01 × 10^−6^	Protein of unknown function involved in RNA metabolism
YDR406W	*PDR15*	14.2	3.31 × 10^−6^	Plasma membrane ATP binding cassette (ABC) transporter
YFL057C	*AAD16*	13.7	1.51 × 10^−5^	Putative aryl-alcohol dehydrogenase
YOL149W	*DCP1*	13.5	3.61 × 10^−5^	Subunit of the Dcp1p-Dcp2p decapping enzyme complex
YDR171W	*HSP42*	13.5	1.19 × 10^−3^	Small heat shock protein (sHSP) with chaperone activity
YIL101C	*XBP1*	12.3	3.01 × 10^−5^	Transcriptional repressor
YHR139C	*SPS100*	12.3	1.95 × 10^−7^	Protein required for spore wall maturation
YGR213C	*RTA1*	12.1	1.04 × 10^−8^	Protein involved in 7-aminocholesterol resistance
YEL039C	*CYC7*	11.8	4.14 × 10^−8^	Cytochrome c isoform 2
YIL056W	*VHR1*	10.5	4.95 × 10^−7^	Transcriptional activator
YCL026C-B	*HBN1*	10.5	8.33 × 10^−6^	Protein of unknown function
YOL123W	*HRP1*	10.4	2.81 × 10^−6^	Subunit of cleavage factor I
YHL036W	*MUP3*	9.5	6.44 × 10^−7^	Low affinity methionine permease
YKR076W	*ECM4*	9.4	4.12 × 10^−7^	Omega class glutathione transferase
YLR108C	*-*	9.1	3.50 × 10^−7^	Protein of unknown function
YER054C	*GIP2*	8.9	1.55 × 10^−7^	Putative regulatory subunit of protein phosphatase Glc7p
YOR298W	*MUM3*	8.9	3.49 × 10^−6^	Protein of unknown function involved in outer spore wall organization
YHL024W	*RIM4*	8.6	4.31 × 10^−8^	Putative RNA-binding protein
YMR169C	*ALD3*	8.3	6.99 × 10^−3^	Cytoplasmic aldehyde dehydrogenase
YOR028C	*CIN5*	8.2	2.47 × 10^−7^	Basic leucine zipper (bZIP) transcription factor of the yAP-1 family
YGR088W	*CTT1*	8.1	3.34 × 10^−6^	Cytosolic catalase T
YER103W	*SSA4*	8.0	1.02 × 10^−5^	Heat shock protein member of the HSP70 family
YER185W	*PUG1*	7.5	1.07 × 10^−5^	Plasma membrane protein involved in protoprophyrin and heme transport
YER053C-A	*-*	7.2	1.56 × 10^−4^	Protein of unknown function
YOR152C	*ATG40*	7.2	3.92 × 10^−5^	Autophagy receptor
YDL204W	*RTN2*	6.7	1.74 × 10^−6^	Reticulon protein
YOR065W	*CYT1*	6.6	4.43 × 10^−6^	Cytochrome c1
YJL051W	*IRC8*	6.6	5.68 × 10^−5^	Bud tip localized protein of unknown function
YLR329W	*REC102*	6.5	4.83 × 10^−6^	Protein involved in early stages of meiotic recombination
YKR077W	*MSA2*	6.4	6.97 × 10^−6^	Putative transcriptional activator
YHR138C	*-*	6.1	7.19 × 10^−3^	Protein of unknown function
YPL201C	*YIG1*	6.0	4.46 × 10^−7^	Protein that interacts with glycerol 3-phosphatase
YDL025C	*RTK1*	6.0	3.61 × 10^−2^	Putative protein kinase
YOR178C	*GAC1*	5.9	9.69 × 10^−4^	Regulatory subunit for Glc7p type-1 protein phosphatase (PP1)
YFL016C	*MDJ1*	5.8	4.14 × 10^−5^	Co-chaperone member of the HSP40 (DnaJ) family of chaperones
YFL030W	*AGX1*	5.8	1.51 × 10^−6^	Alanine:glyoxylate aminotransferase (AGT)
YKL086W	*SRX1*	5.8	5.28 × 10^−5^	Sulfiredoxin
YOR328W	*PDR10*	5.8	1.96 × 10^−6^	ATP-binding cassette (ABC) transporter
YPR151C	*SUE1*	5.6	1.71 × 10^−7^	Protein required for degradation of unstable forms of cytochrome c
YLL026W	*HSP104*	5.5	4.85 × 10^−2^	Disaggregase
YGR243W	*MPC3*	5.5	5.30 × 10^−5^	Highly conserved subunit of the mitochondrial pyruvate carrier (MPC)
YKL093W	*MBR1*	5.5	2.19 × 10^−5^	Protein involved in mitochondrial functions and stress response
YNL036W	*NCE103*	5.5	5.13 × 10^−5^	Carbonic anhydrase
YNL008C	*ASI3*	5.5	1.69 × 10^−5^	Subunit of the nuclear inner membrane Asi ubiquitin ligase complex
YLR343W	*GAS2*	5.5	4.37 × 10^−6^	1,3-beta-glucanosyltransferase
YGR223C	*HSV2*	5.4	1.69 × 10^−6^	Phosphatidylinositol 3,5-bisphosphate-binding protein
YER060W-A	*FCY22*	5.2	1.17 × 10^−5^	Putative purine-cytosine permease
YNL155W	*CUZ1*	5.2	1.90 × 10^−3^	Protein with a role in the ubiquitin-proteasome pathway
YHL021C	*AIM17*	5.2	1.36 × 10^−4^	Putative protein of unknown function
YHR199C	*AIM46*	5.2	1.08 × 10^−5^	Putative protein of unknown function
YGR281W	*YOR1*	5.1	2.18 × 10^−3^	Plasma membrane ATP-binding cassette (ABC) transporter
YGL010W	*MPO1*	5.1	3.53 × 10^−6^	Protein involved in metabolism of phytosphingosine

* Fold change (FC) refers to the fold induction of the genes as compared to the untreated control.

**Table 4:** Functional gene groups induced by the separated or combined exposure to CIT and OTA.

**CIT**	***p*-value**
Gene Ontology Group	
Oxidation-reduction process	1.8 × 10^−13^
Cell response to oxidative stress	2.2 × 10^−9^
Glutathione metabolic process	1.8 × 10^−6^
Drug transport	1.3 × 10^−5^
Response to reactive oxygen species	1.3 × 10^−4^
**OTA**	***p*-value**
Gene Ontology Group	
Single organism developmental process	2.2 × 10^−8^
Oxidation-reduction process	2.0 × 10^−7^
Cell differentiation	3.0 × 10^−6^
Developmental process involved in reproduction	5.4 × 10^−6^
Sporulation	1.6 × 10^−5^
Cell response to oxidative stress	5.4 × 10^−3^
**CIT + OTA**	***p*-value**
Gene Ontology Group	
Oxidation-reduction process	1.7 × 10^−7^
Drug transport	1.3 × 10^−5^
Cell response to oxidative stress	3.1 × 10^−4^
Spore wall assembly	1.4 × 10^−3^
Single organism developmental process	4.2 × 10^−3^
